# Next Generation Sequencing Based Multiplex Long-Range PCR for Routine Genotyping of Autoinflammatory Disorders

**DOI:** 10.3389/fimmu.2021.666273

**Published:** 2021-06-09

**Authors:** Ferhat Guzel, Micol Romano, Erdi Keles, David Piskin, Seza Ozen, Hakan Poyrazoglu, Ozgur Kasapcopur, Erkan Demirkaya

**Affiliations:** ^1^ Department of Research and Development, Gentera Biotechnology, Istanbul, Turkey; ^2^ Department of Paediatrics, Division of Paediatric Rheumatology, Schulich School of Medicine & Dentistry, University of Western Ontario, London, ON, Canada; ^3^ Department of Epidemiology and Biostatistics, Schulich School of Medicine & Dentistry, University of Western Ontario, London, ON, Canada; ^4^ Department of Paediatrics, Division of Paediatric Rheumatology, Hacettepe University, Ankara, Turkey; ^5^ Department of Paediatrics, Division of Paediatric Rheumatology, Erciyes University, Kayseri, Turkey; ^6^ Department of Paediatrics, Division of Paediatric Rheumatology, Cerrhapasa Medical School, Istanbul University, Istanbul, Turkey

**Keywords:** autoinflammation, next generation sequencing - NGS, long range PCR, multiplex, genetic diagnosis

## Abstract

**Background:**

During the last decade, remarkable progress with massive sequencing has been made in the identification of disease-associated genes for AIDs using next-generation sequencing technologies (NGS). An international group of experts described the ideal genetic screening method which should give information about SNVs, InDels, Copy Number Variations (CNVs), GC rich regions. We aimed to develop and validate a molecular diagnostic method in conjunction with the NGS platform as an inexpensive, extended and uniform coverage and fast screening tool which consists of nine genes known to be associated with various AIDs.

**Methods:**

For the validation of basic and expanded panels, long-range multiplex models were setup on healthy samples without any known variations for MEFV, MVK, TNFRSF1A, NLRP3, PSTPIP1, IL1RN, NOD2, NLRP12 and LPIN2 genes. Patients with AIDs who had already known causative variants in these genes were sequenced for analytical validation. As a last step, multiplex models were validated on patients with pre-diagnosis of AIDs. All sequencing steps were performed on the Illumina NGS platform. Validity steps included the selection of related candidate genes, primer design, development of screening methods, validation and verification of the product. The GDPE (Gentera) bioinformatics pipeline was followed.

**Results:**

Although there was no nonsynonymous variation in 21 healthy samples, 107 synonymous variant alleles and some intronic and UTR variants were detected. In 10 patients who underwent analytical validation, besides the 11 known nonsynonymous variant alleles, 11 additional nonsynonymous variant alleles and a total of 81 synonymous variants were found. In the clinical validation phase, 46 patients sequenced with multiplex panels, genetic and clinical findings were combined for diagnosis.

**Conclusion:**

In this study, we describe the development and validation of an NGS-based multiplex array enabling the “long-amplicon” approach for targeted sequencing of nine genes associated with common AIDs. This screening tool is less expensive and more comprehensive compared to other methods and more informative than traditional sequencing. The proposed panel offers advantages to WES or hybridization probe equivalents in terms of CNV analysis, high sensitivity and uniformity, GC-rich region sequencing, InDel detection and intron covering.

## Introduction

As a term, autoinflammation was used to define the diseases not accompanied with high titer of antibodies or T cells. Now it has been understood that these diseases are caused by mutations in genes regulating the innate immune responses (1). Cells that are mediating the pathogenesis of hereditary autoinflammatory diseases (AIDs) are the cells of the innate immune system such as dendritic cells, neutrophils, monocytes and macrophages (2). The diagnosis of AIDs is generally based on the clinical manifestations, symptoms and other biochemical parameters. A detailed clinical history and physical examination are the first steps in the diagnosis and management of autoinflammatory diseases in childhood (3–5). Some of these diseases occur soon after birth and can be fatal if diagnosis and treatment is delayed or unavailable. The differential diagnosis will be maintained so that the treatment modalities can be established before the disease progresses with the help of the genetic screening service. Specific clinical manifestations and genetic analysis are significant for making a differential diagnosis. However, many patients share similar clinical symptoms/features and 50% of patients do not have confirmation by molecular genetic testing (6, 7). Genetic analysis of patients with AIDs allows early and accurate diagnosis and the administration of appropriate treatments. Molecular genetics has greatly contributed to correct diagnosis, especially in atypical presentations (1, 8). These patients are on costly drugs because of empirical treatment modalities due to lack of genetic diagnosis and many patients are using off label or not able to use medications due to not being able to detect genetic mutation. Mutational screening may not be comprehensive related to the used techniques (Sanger Sequencing, Strip Assay etc.), partial gene screening, or screening just known genes and/or mutational hotspots or a subset of coding portions.

During the last decade, remarkable progress with massive sequencing has been made in the identification of disease-associated genes for AIDs using the next generation sequencing technologies (NGS) (1, 8–10). NGS has become an instrumental technology for finding single-gene defects with a comprehensive approach in undiagnosed patients with early onset symptoms (11–14). NGS has advanced the field of autoinflammation by identifying disease-causing genes that point to pathways not known to regulate cytokine signaling or inflammation. Unfortunately, it is still not available for use in routine practice due to great expense in many countries (15). Accurate diagnosis of AIDs is essential to access for the treatment. Overlapping disease manifestations provoke genetic testing among AIDs is the unique way for the diagnosis. International group of experts recently published a guideline and suggested sequencing the 8 genes at a minimum, and if possible additional AIDs genes from the list referenced in Infevers (16). According to this guideline, an ideal genetic screening method should give information about SNVs, InDels, Copy Number Variations (CNVs), GC rich regions, mosaicism and it must be deep sequencing (1000−10,000×). However, there is no tool that currently provides this data for both the clinician and geneticist. Our aim was to develop and validate a molecular diagnostic method in conjunction with NGS platform as an inexpensive, extended and uniform coverage and fast screening tool which consists of nine genes known to be associated with various AIDs.

## Material and Methods

### Sample Collection and DNA Extraction

DNA extraction from blood samples was performed using the QIAamp DNA mini kit (Qiagen, Germany). The concentration and purity of the DNA were measured by a spectrophotometric method (NanoDrop 2000c, Thermo Scientific) at 260/280 nm wave length (17). The DNA was isolated at 25 ng/µl concentration and stored to use at 4°C.

### Primer Design, Multiplex PCR Amplification

**Figure 1:**
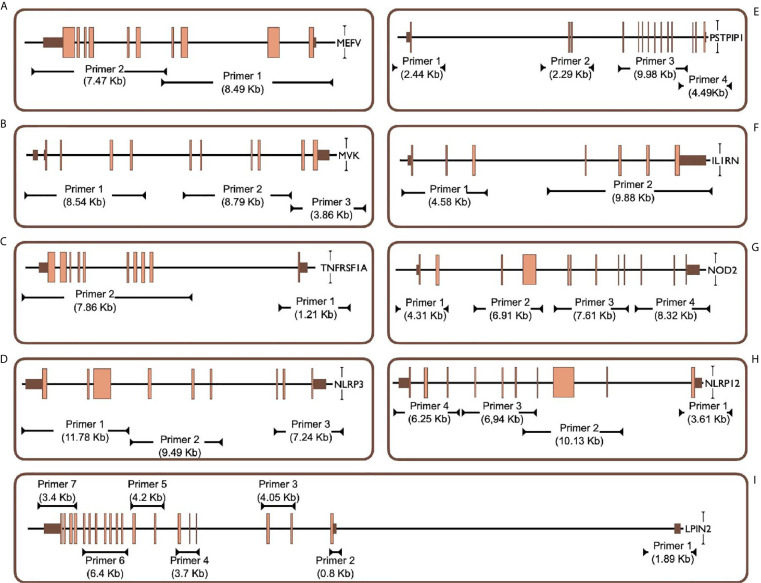
Targeted PCR regions outline in 9 gene. **(A)** MEFV, Mediterranean Fever, **(B)** MVK, Mevalonate Kinase **(C)** TNFRSF1A, Tumor Necrosis Factor Receptor Superfamily Member 1A, **(D)** NLRP3, NLR Family Pyrin Domain Containing 3, **(E)** PSTPIP1, Proline-Serine-Threonine Phosphatase Interacting Protein 1, **(F)** IL1RN, Interleukin-1 Receptor Antagonist, **(G)** NOD2, Nucleotide-binding Oligomerization Domain-containing Protein 2, **(H)** NLRP12, NLR Family Pyrin Domain Containing 12, **(I)** LPIN2, Phosphatidate Phosphatase LPIN2. Brown and orange boxes indicate UTRs and exons, respectively.

Two different multiplex panels were designed as basic and expanded panels. The basic panel is a part of the expanded panel. The basic panel contains MEFV, MVK, TNFRSF1A and NLRP3 genes’ CDS, UTR and some intronic regions. The expanded panel contains PSTPIP1, IL1RN, NOD2, NLRP12 and LPIN2 in addition to the basic panel (**Table 1**). The regions covered by the panels are shown in **Figure 1**. First, gene regions were determined and primers were designed to cover all coding sequences (CDS) and UTRs of genes by using NCBI Primer Blast (18). The Long PCR method was chosen to reduce the number of reactions. With this method, amplicons up to 11.78 Kb were obtained. Each of the primers was tested with healthy control DNAs. For multiplex PCR amplification, primers were designed to minimize primer-dimer formation. Attention was paid to ensure that Tm degrees were close. Reaction contents and conditions were also optimized in this context. The gene regions in the basic panel were amplified in one tube while the expanded panel regions were amplified in four tubes. The 25 µl multiplex PCR amplification reaction volume contained 1,25 unit of LA Taq Hot-Start DNA Polymerase, 1,25 unit of PrimeSTAR GXL DNA Polymerase (TaKaRa Bio, Shiga, Japan), 2.5 µl of 10X LA PCR Buffer II (Mg2+ plus), 4 µl of dNTP mixture (2.5 mM each), 5 µl of Betaine (Sigma), 1 µl (0.2 µM each) of primer mixture (**Table 2**), 1-25 ng of template genomic DNA and the rest ddH20. After determining the appropriate amounts of DNA input and PCR components, the PCR cycle and running parameters were adjusted for effective amplification. The cycling parameters are as follows: initial denaturation at 95°C for 3 min, followed by 34 cycles of 30 second denaturing at 95°C, 40 s annealing at 58°C, and 12 min extension at 68°C. A final extension at 72°C for 12 min concludes the PCR. The PCR reactions were performed using C1000 Thermal Cycler (Bio-Rad). Amplification was seen as a single band in gel electrophoresis. A 1 Kb DNA ladder was used to assess band size. PCR products were purified by Agencourt AMPure XP (Beckman Coulter) magnetic beads and quantified by Qubit 2.0 Fluorometer (Thermo Fisher) with dsDNA BR and HS Reagent Kit.

**Table 1:** Expanded panel content and covered region percentage in exons and introns.

Disease	Gene	Chr	Protein	Inheritance	Gene Length (bp)	Number of Exon	Exon Length (bp)	Intron Length (bp)	Total Amplicon Length (bp)	Exon Coverage Percent	Intron Coverage Percent
FMF	MEFV	16	Pyrin	AR	14.750	10	3.506	11.244	15.962	100%	100%
MKD/HIDS	MVK	12	Mevalonate kinase	AR	24.332	11	2.833	21.499	21.205	100%	85%
TRAPS	TNFRSF1A	12	TNF-R1	AD	13.306	10	2.171	11.135	9.080	100%	62%
CAPS	NLRP3	1	NLRP3	AD	32.645	9	3.545	29.100	28.529	100%	85%
PAPA	PSTPIP1	15	PSTPIP1	AD	42.350	15	1.941	40.409	18.769	100%	41%
DIRA	IL1RN	2	IL1Ra	AR	16.124	4	1.814	14.310	14.470	100%	88%
Blau Syndrome	NOD2 (CARD15)	16	NOD2	AD	33.696	12	4.414	29.282	27.155	100%	77%
FCAS2	NLRP12	19	NLRP12	AD	30.661	10	3.550	27.111	26.953	100%	79%
Majeed Syndrome	LPIN2	18	Phosphatidate phosphatase LPIN2	AR	96.322	20	6.318	90.004	24.526	100%	20%

MEFV, Mediterranean Fever; MVK, Mevalonate Kinase; TNFRSF1A, Tumor Necrosis Factor Receptor Superfamily Member 1A; NLRP3, NLR Family Pyrin Domain Containing 3; PSTPIP1, Proline-Serine-Threonine Phosphatase Interacting Protein 1; IL1RN, Interleukin-1 Receptor Antagonist; NOD2, Nucleotide-binding Oligomerization Domain-containing Protein 2; NLRP12, NLR Family Pyrin Domain Containing 12; LPIN2, Phosphatidate Phosphatase LPIN2; AR, Autosomal Recessive; AD, Autosomal Dominant.

**Table 2:** Primer Sequence and Multiplex Volumes.

Multiplex Tube	Amplicon Name	Primer Sequence (3’-5’)	Tm (UCSC)	Amplicon Length (bp)	Volume (µl)
Multiplex Tube 1	MEFV-1F	CCTCACTTGCCTTCTTGGG	60,8	8490	2,5
	MEFV-1R	AGGTTGCTCTCTACCATCTTCT	56,4		2,5
	MEFV-2F	TAACCAGCAGCCAAGGGTAAG	61,5	7472	1
	MEFV-2R	AAGGGAGAATCGGGAATAAGACA	62,3		1
	MVK-1F	ACTCACCTGTCCCCGTCTT	59,5	8540	1,5
	MVK-1R	CATCTAACCTGCTGCCCTCT	59,5		1,5
	MVK-2F	CACCTCTGCCCGTTCTTCTT	61,7	8797	1
	MVK-2R	CTGGGTCTGTCTCCTGCTTG	61		1
	MVK-3F	ACAAGCAGGAGACAGACCCA	60,9	3868	0,6
	MVK-3R	GAAGACAACAGCAGGGAAGG	59,8		0,6
	TNFRSF1A-1F	TTACAGGAACCCCAGGAGACA	61,8	1219	1,5
	TNFRSF1A-1R	ACTTCACCAGCCGCCAAAA	64		1,5
	TNFRSF1A-2F	CCTGAGACTGCAAAGCACAC	59,6	7861	1
	TNFRSF1A-2R	GAGGGAATGTGGTGGTGGAA	63,1		1
	NLRP3-1F	CCTGCCACATACCAGCCATT	63,2	11783	2,5
	NLRP3-1R	TCTCCACCTTCCACCTCACT	59,7		2,5
	NLRP3-2F	GAGTGAGGTGGAAGGTGGAG	59,7	9499	2,5
	NLRP3-2R	AAGAGCAGGTGATACAGGGAA	58,8		2,5
	NLRP3-3F	AGAGAGGTGGACAGAAAGGG	58,3	7247	2
	NLRP3-3R	GTGTTTGTAGCAGGAAGGCA	58,9		2
Multiplex Tube 2	NOD2-2F	ATCTCGCCTCCTGGGTTGAT	63,2	6910	1
	NOD2-2R	CTCGGTGCTCCCACACTTAG	60		1
	NOD2-3F	CAAGAGGAGTGGCAGACAGG	61	7612	1
	NOD2-3R	TCACCAAACCAGCAAACCCA	64,7		1
	NLRP12-1F	TCCAAGAGTGCTAAGGAGGC	59,6	3618	2
	NLRP12-1R	GACCATCATCCTGCCTACCG	62,8		2
	NLRP12-2F	CAGAGCTGACAAGGGAGGA	59,1	10137	2
	NLRP12-2R	TGGGGTGGAAAAGAGGAGAA	61,9		2
	NLRP12-3F	TGGGGATCAGTCACAAAGGT	60,4	6940	1
	NLRP12-3R	AGGCAAAGAGGGGACAGAGA	61,3		1
	NLRP12-4F	CGTGGGTAGAAGTGCTCAAA	58,9	6258	1
	NLRP12-4R	ACTATGTTCCGATGCAGCCA	61,6		1
	IL1RN-1F	GTAACTGGAAGCGGGATGGA	62,3	4581	1
	IL1RN-1R	TAAGGCAGCAGGACAGGTTT	59,9		1
	IL1RN-2F	GTTGGTTGGAAGATGTGTTGGT	61,1	9889	1
	IL1RN-2R	TTTGCTGCCTTGCCTGTTTC	63,6		1
Multiplex Tube 3	PSTPIP1-1F	CTGGGAGGTGATGGGAAGGA	64,6	2440	1
	PSTPIP1-1R	ACTGAGGCTTGGAGACAGAAA	59,1		1
	PSTPIP1-2F	CTGGAGTGTGCGTGACCTT	59,9	2296	1
	PSTPIP1-2R	CTGTTAGGGTGGCTGTGTCC	60,6		1
	PSTPIP1-3F	TCCTCTGACCCTTGGCTTCT	61,3	9984	1
	PSTPIP1-3R	CACTCCTTTCTGCCCCTTCC	63,3		1
	PSTPIP1-4F	CCACTCTTCAGCCACCCTTC	62,1	4049	1
	PSTPIP1-4R	ACCTTTGCCCACGCACTT	61,7		1
	NOD2-1F	CTTCCCCTCCTCTCCTGTCT	59,8	4311	1
	NOD2-1R	GCCACCACACACTTCCTCT	58,7		1
	NOD2-4F	TTACATTGAGAGCCCTTGGAGT	60,1	8322	2
	NOD2-4R	TGGAGCCACTTTGAGGGAATC	63,7		2
	LPIN2-1F	AGCCTCTCTGTCCACTTCTAAC	56,8	1899	1
	LPIN2-1R	TTCCAAACCACTGCCTACCAA	62,7		1
Multiplex Tube 4	LPIN2-2F	GCAGGAGGTCAGGGTTCTTT	60,6	802	1
	LPIN2-2R	TTCAGTTTCCCTTTCCCTTGA	60,9		1
	LPIN2-3F	CTGAACCGTGTGAGGTGAGG	61,7	4052	1
	LPIN2-3R	GAAACTGCCTTTGCTGCTTG	61,1		1
	LPIN2-4F	TTTATAGTGGGTGGCATTGGTG	61,8	3717	1
	LPIN2-4R	CAAGAGACAGCAGTGTGCAAG	59,8		1
	LPIN2-5F	CCTCGTTCCCAATGCAGGT	63,4	4206	1
	LPIN2-5R	GCCACAGGCCAAACTGAGAA	63,6		1
	LPIN2-6F	GGGAAGCCCTCATTCACTCT	60,6	6439	1
	LPIN2-6R	GCTCCCACACCATCAACAGG	63,9		1
	LPIN2-7F	TAAGGGCTCGTGGAGTTGTC	60,3	3411	1
	LPIN2-7R	GGCGTTTGTGGGTTCCTAAT	61,1		1

### NGS Using Illumina NextSeq 500 System

Barcoded library PCR products were prepared with Nextera XT Sample Prep Kit (Illumina) according to manufacturer’s protocol for > 500 bp amplicon read. 1 ng total multiplex PCR products were used for the preparation of each DNA library. PCR products were enzymatically fragmented with Nextera XT kit (19). Each DNA library was tagged with unique index by 12 cycles of PCR. After cleaning up and normalization steps, all libraries were pooled. Sequencing was performed using the NextSeq 500 Sequencing System (Illumina) with 300 cycle Mid-Output Kit.

### Bioinformatics

Sequencing data was analyzed for rare pathogenic variants that might be associated with the disease. Gentera Data Processing Engine (GDPE) [Gentera (20), Turkey], an easy-to-use automatic pipeline, was used for analyzing genomes. GDPE provides high accuracy variant detection by using different algorithms. A sample sheet and raw data were used as an input. The 5 ‘and 3’ ends of this DNA sequence data are trimmed to certain lengths considering the quality parameters. The DNA sequence data of targeted genes are aligned with using BWA (21) based on the reference human genome sequence (GRCh38). After the alignment, the following steps were followed with using GATK (22) algorithm. Realignment in InDel regions, recalibrating the quality score, parameter optimizations for variations, variant annotation, filtering variants according to Strand Bias status by taking the upper limit (20%), eliminating unreliable (<15%) variations according to the percentage of variation detected. FastQC (23) was used to evaluate the quality of data. Raw VCF file annotated with Annovar (24). The dbSNP150 (25) database was used to determine SNP annotations, amino acid and nucleotide changes and locations in the final report. “Sorting Intolerant from Tolerant” [SIFT (26)] and “Polymorphism Phenotyping” [PolyPhen (27)] applications were used to evaluate the possible impact assessment of variations on proteins. CNV analysis was performed with coverage-based CNVpytor (a python extension of CNVnator) to determine copy number and large structural variations (28). CNVpytor refined the data with multiple-bandwidth partitioning and GC correction approaches.

#### Verifications of the Singleplex and Multiplex Models in Healthy Controls

**Figure 2:**
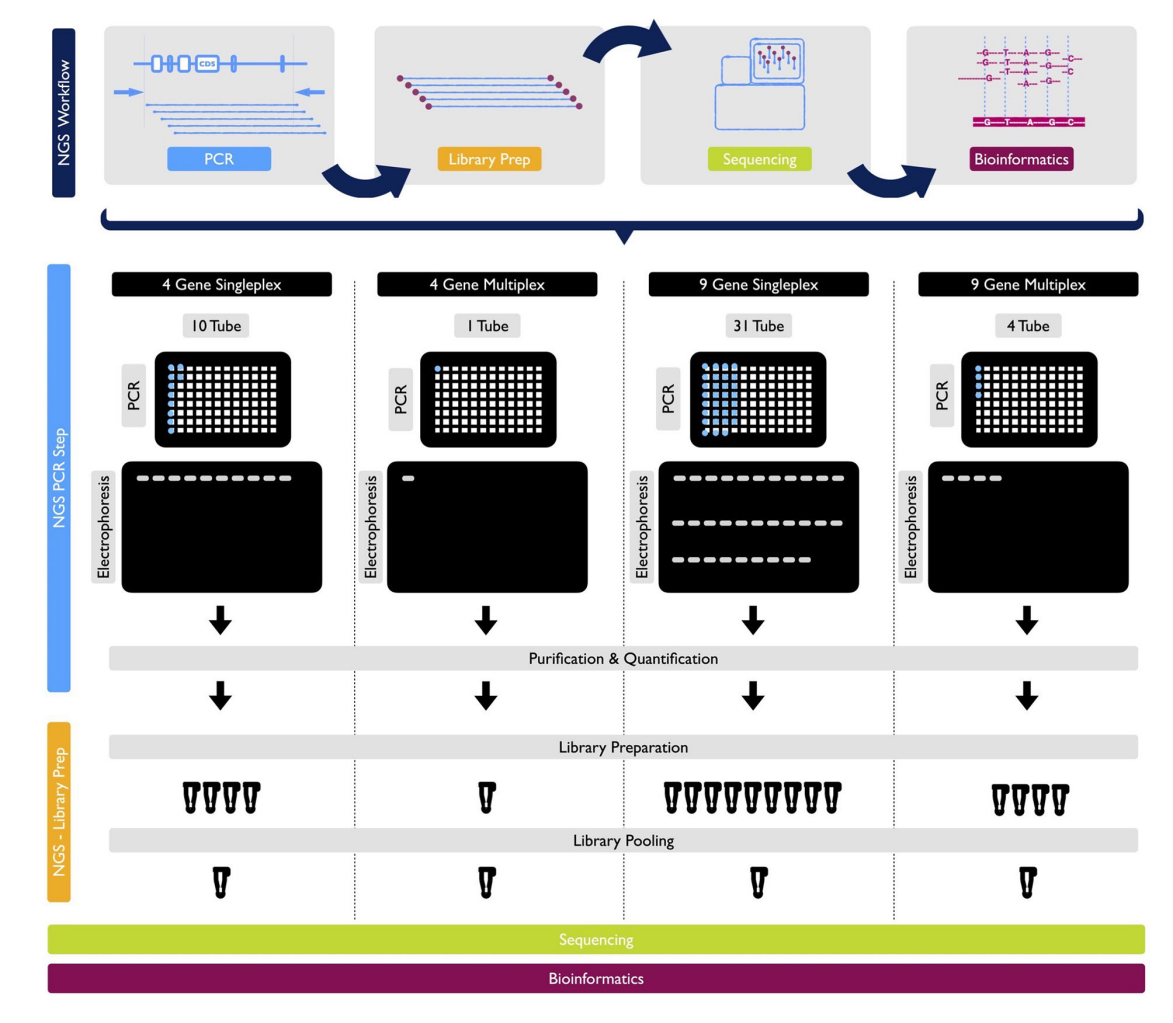
NGS Workflow and detailed protocols for 1 sample by PCR, library prep, sequencing and bioinformatics for 4-gene singleplex, basic multiplex panel, 9-gene singleplex and expanded multiplex panel, respectively.

With the primers designed as described, a trial study was carried out without any known variations on the genes being sequenced. Each of the 9 genes was sequenced separately in different DNA with the singleplex model and visualized using gel electrophoresis. The singleplex PCR products controlled on the gel were sequenced as described in the NGS system. The same procedure was performed for basic and expanded panels on healthy controls. Multiplex PCR primers and conditions were prepared as described. They were visualized on gel electrophoresis (**Figure 2**). After PCR products were sequenced as described in NGS system, optimization was done by decreasing the number of amplicons with high reading in NGS results, increasing the number of amplicons with low reading and changing reaction conditions.

#### Analytical Validations of the Multiplex Models in Known Mutation Carriers

Genomic DNA samples from patients with AIDs who had already been found to carry at least one variation in one of the known causative genes previously tested through Sanger sequencing or Strip Assay Kits (ViennaLab) were collected. These variation-positive patients were taken into consideration for developing an NGS-based diagnostic protocol.

#### Clinical Validations of the Multiplex Models in Undiagnosed Patients

Patients underwent screening history, physical examination, and laboratory evaluation, in the outpatient department in ten different pediatric and adult rheumatology centers where these groups of patients are mainly followed up in Turkey and pre-diagnosed with FMF, MKD (HIDS), TRAPS, CAPS, PAPA, DIRA, Blau, FCAS2 and Majeed syndrome. Initially, data was collected on their clinical parameters such as presence and duration of fever, frequency of attacks, abdominal pain, age of onset, organ system involvement, the presence of visible lesions (rashes, purpura, nodules etc.). Molecular diagnostics were also considered based on their clinical presentations and response to therapies. Blood samples from previously consented patients and, in some cases, unaffected family members, were collected to extract DNA, and perform NGS analysis with basic and expanded panels (**Figure 2**).

## Results

### Verifications of the Singleplex and Multiplex Models in Healthy Controls

**Figure 3:**
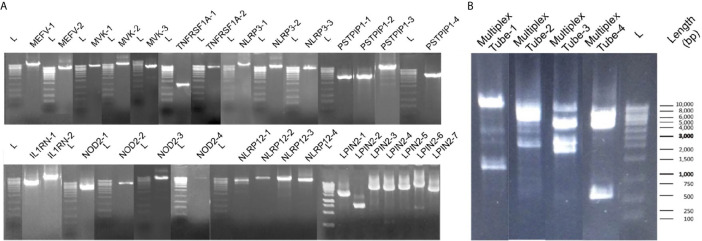
Band patterns of the PCR products. **(A)** Singleplex 31 amplicons band patterns of MEFV, MVK, TNFRSF1A, PSTPIP1, IL1RN, NOD2, NLRP12 and LPIN2 are shown respectively. L label indicates the 1 kb sized DNA ladder. **(B)** The multiplex band patterns of the MEFV, MVK, TNFRSF1A and NLRP3 are shown in Multiplex Tube-1. PSTPIP1, IL1RN, NOD2, NLRP12 and LPIN2 patterns are shown in Multiplex Tube 2-4. L label indicates the 1 kb sized DNA ladder.

For singleplex optimization, the PCR procedure was applied to all primer pairs separately on 9 healthy control DNA samples. In gel electrophoresis, the clear unique-band appearance reflects that the targeted PCR product is amplified (**Figure 3A**). Basic and expanded panels were developed after optimization of primer composition and PCR conditions with using specific primer sets. Ten healthy control DNA samples were screened with the basic panel, similar PCR product patterns were observed with singleplex electrophoresis. Eight PCR products longer than 7.5 Kb were observed as single thick band. Two bands of 3.8 and 1.2 kb were also observed separately (**Figure 3B**). Adequate quality was observed in all regions covered by the primers. Two healthy control DNA samples were tested with expanded panel and bands were observed in 0.8 kb and 10 kb range. In total, 21 control samples were sequenced, and no nonsynonymous variation was detected. A total of 107 synonymous variations were detected. 55% of these variations were found on the MEFV (n=13) and 25% on the NLRP3 (n=13). Of the 130 UTR variations detected, 48% were on MEFV and 30% were on NLRP3. In addition, 16% of 1221 intronic variations were found on MEFV, 17% on MVK (n=13) and 42% on NLRP3 (**Table 3**).

**Table 3:** Detected variant alleles at verification, analytical validation and clinical validation steps in patients with AID.

	Variant Type		AA Change	dbSNP Code	Exon number	Number of Variant Allele at Verification (n=21)	Number of Variant Allele at Analytical Validation (n=10)	Number of Variant Allele at Clinical Validation (n=46)	Total Number of Variant Allele
MEFV	Nonsynonymous	SNV	E148Q	rs3743930	2			3	50
			G304R	rs75977701	2			1	
			R202Q	rs224222	2		1	23	
			P369S	rs11466023	3			3	
			R408Q	rs11466024	3			3	
			M680I*	rs28940580	10		1	2	
			M694V*	rs61752717	10		1	7	
			V726A	rs289940579	10		1	2	
			A744S	rs61732874	10			2	
	Synonymous	SNV				59	32	170	261
	UTR	SNV				63	22	91	176
		Insertion				12	6	28	46
		Deletion				9	4	18	31
	Intronic	SNV				204	57	619	880
		Insertion				5	4	19	28
		Deletion				40	8	101	149
MVK	Nonsynonymous	SNV	P11L	rs876661001	2			1	17
			S52N*	rs7957619	3		4	12	
	Synonymous	SNV				3	4	20	27
	UTR	SNV				6	5	35	46
		Insertion				3	2	12	17
		Deletion							0
	Intronic	SNV				210	122	698	1030
		Insertion				3	2	25	30
		Deletion				41	23	135	199
TNFRSF1A	Nonsynonymous	SNV	R92Q*	rs4149584	4		1		1
	Synonymous	SNV				8	5	27	40
	UTR	SNV							0
		Insertion						4	4
		Deletion						2	2
	Intronic	SNV				72	30	177	279
		Insertion				3		9	12
		Deletion				7	4	16	27
NLRP3	Nonsynonymous	SNV	Q703K*	rs35829419	3		3	6	9
	Synonymous	SNV				27	20	101	148
	UTR	SNV				40	28	134	202
		Insertion				11	10	38	59
		Deletion				10	5	38	53
	Intronic	SNV				518	254	1650	2422
		Insertion				65	27	163	255
		Deletion				103	44	304	451
PSTPIP1	Nonsynonymous	SNV	A196V*	rs370965231	9		1		1
	Synonymous	SNV						3	3
	UTR	SNV						1	1
		Insertion				1		3	4
		Deletion						1	1
	Intronic	SNV				17		64	81
		Insertion				3	4	9	16
		Deletion				2		3	5
IL1RN	Synonymous	SNV				2	4	5	11
	UTR	SNV				5	6	13	24
		Insertion							0
		Deletion				1		1	2
	Intronic	SNV				65		147	212
		Insertion				3		6	9
		Deletion						3	3
NOD2	Nonsynonymous	SNV	P268S	rs2066842	4		2	3	18
			R676C	rs5743277	4			1	
			G775D*	16:50712235 (GRCh38)	4		1		
			M491L	16:50711382 (GRCh38)	4			3	
			N852S	rs104895467	6			1	
			G908R	rs2066845	8			1	
			V955I*	rs5743291	9		3	3	
	Synonymous	SNV				4	9	16	29
	UTR	SNV				4	12	21	37
		Insertion							0
		Deletion							0
	Intronic	SNV				23	24	92	139
		Insertion				1		4	5
		Deletion				5	4	12	21
NLRP12	Nonsynonymous	SNV	N394K*	rs201241894	3		1		1
	Synonymous	SNV				4	7	15	26
	UTR	SNV				2		2	4
		Insertion							0
		Deletion				1	2	3	6
	Intronic	SNV				78	14	166	258
		Insertion				10	1	2	13
		Deletion				17		13	30
LPIN2	Nonsynonymous	SNV	E601K	rs61735393	14		1		2
		SNV	P348L*	rs34676691	7		1		
	Synonymous	SNV						2	2
	UTR	SNV				10	16	23	49
		Insertion				2	4	3	9
		Deletion							0
	Intronic	SNV				34		60	94
		Insertion				1			1
		Deletion				10		6	16

*Known variations (AA change) in analytical validation of multiplex model.

### Analytical Validations of the Multiplex Models in Known Mutation Carriers:

At this step, 5 patients with known MEFV:M694V, MEFV:M680I, MVK:S52N, TNFRSF1A:R92Q, NLRP3:Q703K/Q703K variations were screened with the basic panel. Another 5 patients with known PSTPIP1:A196V, NOD2:V955I, NOD2:G775D, NLRP12:N394K, LPIN2:P348L variations were screened with the expanded panel. In addition to these nonsynonymous variations, 11 more nonsynonymous allele variants were detected. A total of 81 synonymous variations were detected. 39% of these variations were found on the MEFV (n=10) and 24% on the NLRP3 (n=10). Of the 89 UTR variations detected, 24% were on MEFV and 31% were on NLRP3. In addition, 50% of 501 intronic variations were found on NLRP3 and 24% on MVK (n=10) (**Table 3**).

### Clinical Validations of the Multiplex Models in Undiagnosed Patients

Panels validated with prediagnosed AIDs patients (n=46) who were diagnosed by primary attending physician, reported symptoms which were associated with episodes of the patients with undiagnosed AIDs were screened with panel in this step summarized in **Table 4**. The most common symptoms were fever>38C (82.6%), abdominal pain (52.2%), arthralgia (52.2%). Skin involvement was described in 50% of the patients such an urticarial rash, maculo-papular rash, pseudo-folliculitis, and erysipelas-like rash. The number of episodes in a year were reported as more than 12 (10.9%), 6-12 (34,8%) and between 2 and 6 (54.3%) by the patients or their parents. The duration of episodes in our cohort were 2-5 days in 71.7%, 5-10 days in 8.7% and >10 days in 19.6%.

**Table 4:** Clinical manifestations of the patients.

*Clinical manifestations in 46 patients*			
	n (%)		n (%)
***Constitutional Symptoms***		***Cardiorespiratory***	
Fever (>38C)	38 (82.6%)	Thoracic pain	9 (19.6%)
Headache	10 (21.7%)	Pleurisy	3 (6.5%)
Fatigue	15 (32.6%)	Pericarditis	1 (2.2%)
***Musculoskeletal***		***Lymphoid***	
Skeletal dysplasia	1 (2.2%)	Lymphadenopathy	8 (17.4%)
Skull anomaly	1 (2.2%)	Splenomegaly	7 (15.2%)
Overgrowth patella	1 (2.2%)	***Gastrointestinal***	
Myalgias	18 (39.1%)	Abdominal pain	24 (52.2%)
Arthralgia	24 (52.2%)	Peritoneal adhesion	2 (4.3%)
Arthritis	7 (15.2%)	Constipation	4 (8.7%)
***Mucocutaneous***		Diarrhea	11 (23.9%)
Aphthous stomatitis	6 (13%)	Vomiting	9 (19.6%)
Exudative pharyngitis	5 (10.9%)	***Ocular***	
Cold urticaria	1 (2.2%)	Conjunctivitis	4 (8.7%)
Maculo-papular rash	11 (23.9%)	Keratitis	2 (4.3%)
Urticarial rash	6 (13%)	Periorbital edema	5 (10.9%)
Erysipelas-like rash	2 (4.3%)	Visual loss	1 (2.2%)
Pseudo-folliculitis	2 (4.3%)		

**Figure 4:**
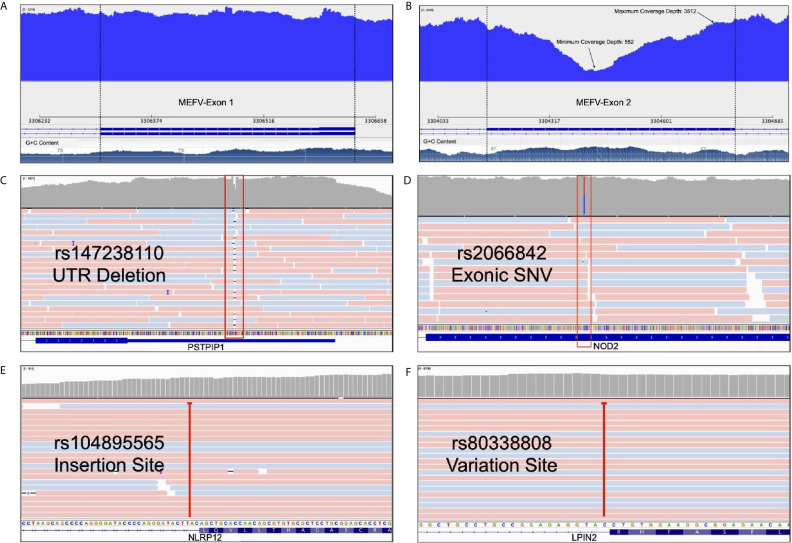
Advantages of our multiplex model. **(A)** Uniform coverage demonstration on MEFV first exon* **(B)** Minimum and maximum coverage depth of the MEFV second exon with high GC content* **(C)** A deletion from PSTPIP1’s UTR as an example of InDel determination. **(D)** A heterozygosis SNV detection of NOD2 with deep sequencing rate **(E)** An intronic NLRP12 insertion site that deeply covered **(F)** An intronic LPIN2 variation site that cause frameshift. *These figures show the change of coverage depth along the exon. G+C content graph can be seen at the bottom.

**Figure 5:**
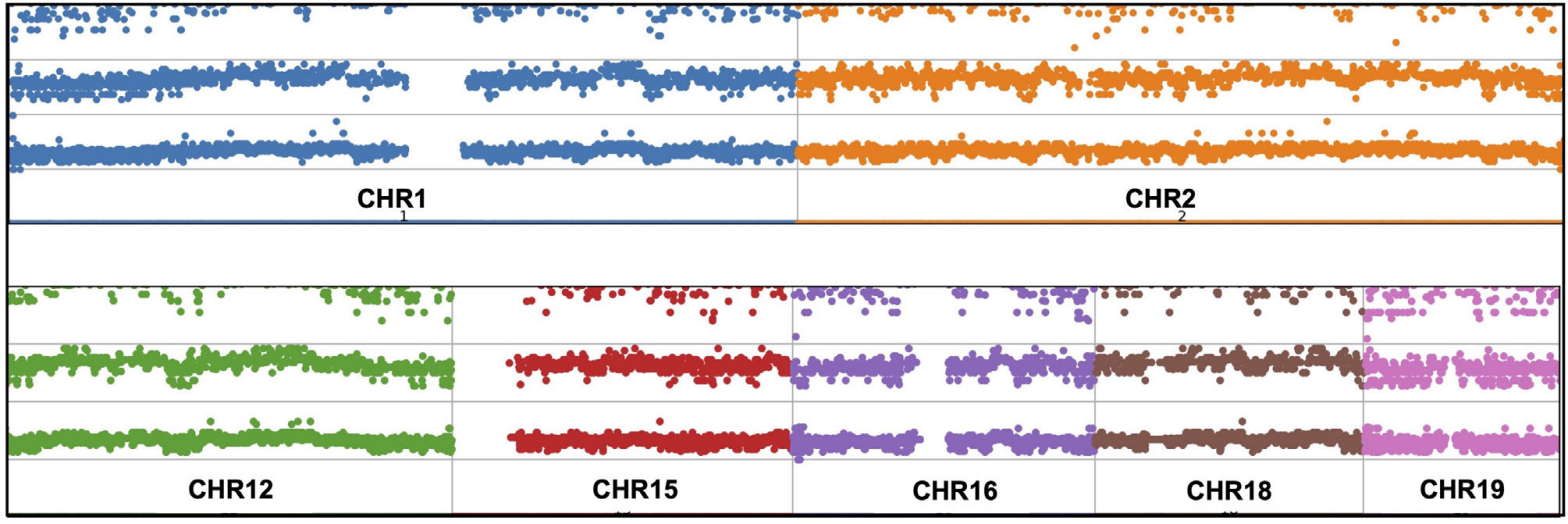
CNV analysis manhattan plot demonstration throughout the sequenced chromosomes.

Expanded panels were tested in 14 samples, basic panels tested in 32 samples according to their possible clinical diagnosis. Primary physicians requested to screen a total of 129 genes from 46 patients with the preliminary diagnosis of FMF (n=18), CAPS (n= 25), MKD (n=19), TRAPS (n=23), Blau (n=10), DIRA (n=9), Majeed (n=9), PAPA (n=8) and NALP12 (n=8). Exonic and intronic variations were detected and grouped according to their variant types and summarized in **Table 3**. A total of 77 nonsynonymous variations were detected. 59% of these were found on MEFV (n=46), 17% were on MVK (n=46), 15% were on NOD2 (n=14) and 7% were on NLRP3 (n=46). A total of 359 synonymous variations were detected and 47% of these variations were found on MEFV and 28% on the NLRP3. Of the 320 UTR variations detected, 28% were on MEFV and 41% were on NLRP3. In addition, 50% of 3673 intronic variations were found on NLRP3, 19% on MVK and 16% on MEFV. Uniform coverage was obtained from exonic, intronic and UTRregions (**Figure 4**). Also, the CNV alteration was not seen in any sample (**Figure 5**).

## Discussion

In this study, we describe the development and validation of NGS-based multiplex array enabling the “long-amplicon” approach, which allows compatibility for both long and short read NGS systems including Illumina (Miseq, Hiseq and Nextseq500), Oxford Nanopore and other NGS platforms for targeted sequencing of the nine genes associated with the most common AIDS. Validity steps included the selection of related candidate genes, primer design, development of screening methods, validation and verification of the product. This screening tool is less expensive and more comprehensive compared to other methods and more informative than traditional sequencing. Regarding the complex clinical and molecular diagnosis for AIDs, it should be emphasized that this diagnostic kit will simultaneously test all known genes and variants.

Our long-range multiplex model is enabled to screen exon-intron boundaries and deep introns. WGS approach shows that there is an augmenting number of pathogenic variants positioned within introns at least 100 bp away from the exon-intron junction (29). Moreover, Genome-Wide Association Studies (GWAS) revealed that many intronic variants have a significant association with diseases (30, 31). Protein translation and expression are affected by deep intronic variants which were observed in cystic fibrosis or collagenopathies (32). Current deep intronic variants which can cause AIDs have not been described. WES, hybridization probe-based sequencing and microarray-based analysis failed to detect intronic variations (29). The relationship between intronic variants and AIDs has been reported. In a patient with periodic fever syndrome, an intronic NLRP12 pathogenic variant (rs104895565) activated a cryptic splice site upstream in exon 3 and caused a frameshift followed by an early stop codon (33). Similarly, an intronic LPIN2 pathogenic variant (rs80338808) reported in a patient with chronic recurrent multifocal osteomyelitis (CRMO) and congenital dyserythropoietic anemia (CDA) caused a frameshift in exon 17 which resulted in early stop codon (34). Our results showed that our panel detects potential intronic variations such as previously described rs104895565 and rs80338808 and able to extend in the light of new information (**Figures 4E, F**). We found the least intron coverage in the LPIN2 gene with 20% and the highest coverage in the MEFV gene (100%). We are able to cover 80% of the intronic regions in six genes among our panel. 

Long-read sequencing instruments perform with high accuracy in detecting small insertions, deletions (InDels) and large complex structural variants compared to short-read systems (35). Our panel is compatible with long read sequencing systems which allow high coverage and more accurate results. Rowczenio et al. performed targeted autoinflammatory panel to investigate the molecular cause of persistent fevers resulting in a 24 nt pathogenic mutation being identified in a patient with TRAPS and confirmed as the first known case of gonosomal TNFRSF1A mosaicism (36). Our panels allow us to detect inDels in exonic, UTR (**Figure 4C**) and intronic regions.

Methods with high specificity and uniform coverage can achieve adequate quality even with a lower sequence data. This also makes sequencing more cost effective. The specificity and uniformity of amplicon-based studies are too high to compare with such WES or targeted hybrid capture based methods (37). Our panels are highly specific to the exons of the genes they contain (**Figure 4D**). We performed *in-silico* analysis and BLAST for our panel to increase specificity not to hit a different region in the human genome. Our NGS results showed that, uniform coverage depth was observed except for GC-rich and repetitive regions (**Figure 4A**). Sequencing difficulties through GC-rich sequences in AIDs related genes (such as MEFV exon 2) underlined as an important concern for the diagnosis (38). We obtained minimum 552X and maximum 3512X coverage depth for MEFV exon 2 with our multiplex panel (**Figure 4B**). To improve GC-rich sequence reading quality, we used betaine in the PCR step (39). Also, PCR conditions are optimized for the best GC-rich sequence amplification. Our results revealed that high quality sequence readings obtained targeted GC-rich regions.

Copy number variations (CNVs), intermediate structural variants, refers to DNA copy number changes between 1 Kb to 5 Mb (40). There are different CNV calling strategies according to analysis type of NGS including paired-end read (41), read-depth (42) and split read (43). Whole exome sequencing (WES) studies require much effort for CNV analysis (44). Nevertheless, previous studies demonstrate the limitation such as low sensitivity and high false positive rates (45). Shinar et al. have highlighted the importance of CNVs in the genetic analysis of patients with AIDs (16). For sensitive CNV analysis, above 1000X average reading depth (46) and uniform sequence coverage are needed (47). In particular, detection of structural variants is a crucial and recommended for diagnosis of the AIDS (16). Mosaicism derives as a result of single nucleotide variations (SNVs) and CNVs and NGS is a useful method for identifying, categorizing, verifying and validating (48). The 1000X over coverage obtained outside of GC-rich and repetitive regions and uniform read depth made our multiplex model suitable for CNV analysis. Attention was paid to ensure that the algorithm chosen for data analysis was suitable for these parameters. However, no major structural changes and copy number changes were encountered (**Figure 5**).

Depending on the method and chemistry used, our panels have some limitations. The panel containing 9 genes does not include all genes related to AIDS. Our effort continues to expand the panel content to increase the number of genes. Focusing on specific regions of the genome reduces the possibility of finding novel variations (49). Compared to WGS, our panel is limited in detecting large structural variants and CNVs (50). Both PCR amplification and library preparation, DNA polymerase is used which causes artifacts. The artifacts cause the base substitution errors between 1/3.200 to 1/300.000 errors/base rates (51). In order to reduce the error caused by DNA polymerase and increase the amplification efficiency, we preferred high compatibility enzyme in our studies (52).

As a conclusion, in this study, we described the development and validation of NGS-based multiplex array enabling the “long-amplicon” approach for targeted sequencing of nine AIDs genes. This screening tool is less expensive and more comprehensive compared to other methods and more informative than traditional sequencing. The proposed panel has an advantage compared to WES or hybridization probe equivalents in terms of CNV analysis, high sensitivity and uniformity, GC-rich region sequencing, InDel detection and intron covering (**Table 5**). Currently, there are 29 genes that have been associated with more than 30 hereditary auto-inflammatory disorders (http://fmf.igh.cnrs.fr/ISSAID/infevers/). The screening tool will be updated from time to time to incorporate the newly discovered genes.

**Table 5:** Comparison of the methods in terms of quality, duration and cost.

		Whole Exome Seq	Hybridization Probe Targeted Seq	Singleplex Long-Range Amplicon		Multiplex Long-Range Amplicon	
				4-Gene	9-Gene	4-Gene	9-Gene
** Hands-on Time Rate for 1 Sample**	PCR	–	–	3X	8X	1X	2X
	Gel Electrophoresis	–	–	3X	5X	0.5X	1X
	Library Preparation	30X	8X	6.5X	6.5X	6.5X	6.5X
	Total	30X	8X	12.5X	19.5X	8X	9.5X
** Coverage**	High Output (120 Gb)	Mod.	High	High	High	High	High
	Mid Output (32 Gb)	Low	High	High	High	High	High
	Low Output (5Gb)	–	Mod.	Mod.	Mod.	Mod.	Mod.
** Region**		Exons	Exons	Exons & Some Introns	Exons & Some Introns	Exons & Some Introns	Exons & Some Introns
** CNV Analysis**		Low	Low	High	High	High	High
** InDels Detection**		Low	Low	High	High	High	High
** Uniform Coverage**		Low	Low	High	High	High	High
** Compatibility with Different Sequencing Platforms**		–	–	+	+	+	+
** Sensitivity**		Low	Low	High	High	High	High
** GC-Rich Content Detection**		Low	Low	High	High	High	High
** Estimated Cost Rate for 1 Sample**	High Output (120 Gb)	35X	24X	10X	15X	7X	8X
	Mid Output (32 Gb)	25X	20X	7X	12X	5X	6X
	Low Output (5Gb)	–	19X	6X	11X	4X	5X

The data in the table were prepared based on 2*150bp long readings and estimated cost calculated based on consumables price.

## Data Availability Statement

All data relevant to the study are included in the article or uploaded as supplementary information. The raw datasets presented in this article are not readily available because of institutional restrictions and patient privacy. Requests to access the datasets should be directed to corresponding author. The semi processed data supporting the conclusions of this article will be made available by the authors, without undue reservation.

## Ethics Statement

The studies involving human participants were reviewed and approved by Cerrahpasa Medical Faculty, Istanbul, Turkey, with the registry number of 83045809/604.01/02-312418/A-31 on 07.10.2015. Written informed consent to participate in this study was provided by the participants’ legal guardian/next of kin.

## Author Contributions

ED and FG coordinated the study, drafted the manuscript analyzed the data. FG and EK performed experiments. FG, DP, and MR performed statistical analysis. ED, SO, HP, and OK participated in writing the manuscript with input from all authors. ED, HP, and OK included patients, provided clinical information and samples. ED revised the manuscript critically for important intellectual content and have given final approval of the version to be submitted for publication. ED as a PI had full access to all of the data in the study and takes responsibility for the integrity of the data and the accuracy of the analysis. All authors contributed to the article and approved the submitted version.

## Conflict of Interest

The authors declare that the research was conducted in the absence of any commercial or financial relationships that could be construed as a potential conflict of interest.
